# Quantitative comparison of lipoprotein fractions derived from human plasma and serum by liquid chromatography-tandem mass spectrometry

**DOI:** 10.1186/1477-5956-8-42

**Published:** 2010-07-29

**Authors:** Lisamarie A Collins, Michael Olivier

**Affiliations:** 1 Biotechnology and Bioengineering Center, Medical College of Wisconsin, Milwaukee, WI, USA; 2 Department of Physiology, Medical College of Wisconsin, Milwaukee, WI, USA

## Abstract

**Background:**

Lipoproteins are complex, globular molecules which play essential roles in the transport and metabolism of cholesterol. Their implication in the development of cardiovascular diseases, such as atherosclerosis, has sustained a great deal of interest in these particles. Their various functions, and their contribution to the development of atherosclerosis, are often attributed to their protein constituents, which vary greatly among the different lipoprotein classes. Recent advances in the field of mass spectrometry have provided insight into the array of proteins associated with low-density lipoproteins (LDLs) and, even more so, with high-density lipoproteins (HDLs). Plasma and serum are the most commonly used samples for the analysis of lipoproteins. Although these lipoprotein sources are unique, it was our goal to determine whether or not their inherent differences would ultimately affect a quantitative analysis of the LDL and HDL proteomes. To this end, we isolated LDL and HDL fractions with fast protein liquid chromatography-size exclusion chromatography (FPLC-SEC) from both human plasma and serum samples from the same individuals. After delipidating these samples, we performed a quantitative proteomic analysis to compare the lipoprotein-associated proteins derived from both plasma and serum.

**Results:**

Although the primary differences between the samples are found in fibrinogen proteins which are removed from serum, it of interest to note that, with respect to LDL-associated proteins, apolipoproteinB-100 was found at significantly higher levels in serum samples. Complement component 3 was found at significantly higher levels in serum-derived HDL fractions. Both of these proteins are known LDL- and HDL-associated proteins, respectively.

**Conclusion:**

Overall, the results from our study indicate that both plasma and serum samples are equally suited for proteomic studies, and provide similar results. These findings are particularly important for studies profiling proteomic differences in lipoprotein particle composition in a variety of disease conditions, including cardiovascular disease.

## Background

Lipoproteins, as their name implies, are complex, globular aggregates of lipid and protein which circulate primarily in plasma/serum, and play a central role in the transport and metabolism of both endogenous and exogenous cholesterol and other lipids. It is a well established fact that, in general, low-density lipoprotein (LDL) levels are directly correlated with the risk for the development of cardiovascular disease, whereas high-density lipoprotein (HDL) levels are inversely correlated with this risk [1,2]. The role that these particles play in the development of atherosclerosis has sustained decades of interest in studies devoted to lipoprotein function and metabolism. As LDL and HDL are composed of roughly twenty-two percent and fifty percent protein, respectively, there is recognition of the fact that the protein component of these particles is largely responsible for carrying out their various functions. Many efforts have been focused on the elucidation of the proteomes (the protein components) of both LDL and HDL [3-7]. Tremendous advances in the field of mass spectrometry have allowed for the identification of a vast array of proteins, many of which were previously not known to associate with lipoproteins. Since they contain a relatively larger protein constituent, it is not surprising that the majority of these lipoproteomic analyses have been aimed at HDLs as opposed to LDLs. Nonetheless, the identification of both LDL- and HDL-associated proteins, and, in particular, the quantitative characterization of their proteomes in disease states, provides tremendous potential for the detection of risk factors and for the development of interventions for the treatment of lipoprotein-associated disorders.

The proteomic studies which have concentrated on HDL have collectively identified over eighty different proteins [3,5-7]. Several of these proteins and enzymes, which likely mediate the atheroprotective functions of HDL, have been found to be altered in cardiovascular and metabolic diseases. A recent shotgun proteomics analysis of HDL has revealed that these particles contain not only proteins involved in lipid metabolism, but also proteins that are involved in complement regulation, inflammation and proteinase inhibition [7]. Vaisar *et al*. also characterized the HDL_3 _subset of patients with established coronary artery disease and found it to be selectively enriched in inflammatory response and complement pathway proteins such as paraoxonase-1 (PON1) and complement component 3 (C3). Activity levels of PON-1, a low-abundance HDL-associated protein, have been found to be decreased in patients with metabolic syndrome [8]. Furthermore, there is evidence that a number of other low-abundance proteins, which may only weakly associate with HDL particles, are altered in potentially proatherogenic conditions. Under inflammatory conditions, it has been shown that both apolipoprotein J (clusterin) and serum amyloid A (SAA) levels increase [9-11].

One of the goals of the HUPO Plasma Proteome Project (PPP), which was piloted in 2002, is to analyze comprehensively the protein constituents of human plasma and serum [12]. As a result of this initiative, several of the challenges associated with studying plasma, serum, and their constituents, such as lipoproteins, have been highlighted. One such challenge is related to the fact that plasma and serum are highly complex samples for proteomic analyses. The dynamic range of proteins found in plasma and serum spans greater than ten orders of magnitude, exceeding even the most sensitive mass spectrometers [13], thereby making the detection of proteins on the low-abundance end of the spectrum more difficult. With this knowledge, several methods for the depletion of the most abundant plasma proteins have been developed. With respect to the analysis of lipoproteins, arguments have been made for the selective enrichment of lipoprotein-specific proteins such as apolipoprotein B-100 (apoB-100) and apolipoprotein A-I (apoA-I) via immunoaffinity chromatography. However, the major concern over the employment of such depletion techniques is related to the loss of potential proteins of interest associated with lipoproteins as we have previously described [14]. Nonetheless, the issue of sensitivity is a concern which can be extended to the field of lipoproteomics.

Plasma and serum, which are very similar in protein composition, are the most commonly used samples for the analysis of lipoproteins. Although serum contains less protein (due to removal of the fibrin clot), the products of *in vitro *proteolysis, which occurs during the clotting process, may alter the protein composition of the serum sample [15]. Consequently, much of the argument favoring the use of plasma over serum in proteomic studies stems from the concern over *in vitro *proteolysis. Although we recognize that plasma and serum are qualitatively unique, it was our goal to determine if their inherent differences affect the quantitative analysis of LDL- and HDL-associated proteins. Given the difficulties in analyzing samples with broadly ranging protein abundances, it will be beneficial to select a sample which will yield the best results with respect to identifying lipoprotein-associated proteins. Following the same reasoning, it would be equally beneficial to know that, if there are no significant differences between the abundances of lipoprotein-associated proteins in serum and plasma, the use of one sample over the other will not substantially bias one's results and conclusions. To this end, we isolated LDL and HDL from plasma and serum collected from healthy volunteers using fast protein liquid chromatography-size exclusion chromatography (FPLC-SEC). After delipidating these samples, we quantified the lipoprotein-associated proteins in plasma and serum. Our results show that the primary differences between the samples are found in the fibrinogen proteins found in plasma which are contained within the fibrin clot and are removed from serum. Therefore, plasma and serum are of equal value for quantitative analyses of either the LDL or HDL proteomes.

## Results

It was the purpose of the present study to determine if there are significant differences between plasma and serum with respect to the proteins identified in the FPLC-SEC-derived lipoprotein fractions. In particular, our goal was to analyze the FPLC-SEC-derived fractions containing LDL and HDL particles. After isolating the lipoprotein particles and performing lipid extractions, the protein constituents of LDL and HDL were analyzed by nano-HPLC electrospray ionization tandem mass spectrometry. We identified proteins unique to the LDL fraction and to the HDL fraction. These results can be found in Tables 1 and 2, respectively. We also compiled a list of proteins which overlap between the two lipoprotein fractions. This list can be found in Table 3. A representative total ion chromatogram for one of the FPLC-derived plasma HDL fractions along with a selected mass spectrum for an identified peptide can be found in Additional File 1.

**Table 1:** Proteins unique to the LDL fraction.

Protein Accession	Protein Description	Plasma Avg. Scan Count ± SEM	Serum Avg. Scan Count ± SEM
P02745	Complement C1q subcomponent subunit A	1 ± 0.63	1.2 ± 0.63
P02746	Complement C1q subcomponent subunit B	1 ± 0.52	2.2 ± 0.95
P02747	Complement C1q subcomponent subunit C	1.7 ± 0.47	3 ± 1.3
P00736	Complement C1r subcomponent	5.7 ± 1.7	3.2 ± 2.6
P09871	Complement C1 s subcomponent	0.33 ± 0.30	0.8 ± 0.48
P20851	C4b-binding protein beta chain	0.67 ± 0.47	1.4 ± 1.2
O43866	CD5 antigen-like	8.3 ± 2.8	12.8 ± 4.2
P00488	Coagulation factor XIII A chain	0.33 ± 0.30	0
O75636	Ficolin-3	0.17 ± 0.24	1.2 ± 0.48
P07359	Platelet glycoprotein Ib alpha chain	0.17 ± 0.24	0.4 ± 0.5
P69905	Hemoglobin subunit alpha	18.2 ± 25 *	1.8 ± 1.4
P68871	Hemoglobin subunit beta	34.5 ± 48 *	5.6 ± 3
Q08380	Galectin-3-binding protein	2.3 ± 1.3	2.2 ± 1.3
Q93074	Mediator of RNA polymerase II transcription subunit 12	0.33 ± 0.47	2.6 ± 1.9
P27918	Properdin	0	0.8 ± 0.6
P07225	Vitamin K-dependent protein S	2.8 ± 1.9	3.4 ± 3.5

The average scan count ± SEM is provided for each protein identified in only the LDL fraction derived from both plasma and serum. (P < 0.001; * significantly greater in plasma;† significantly greater in serum)

**Table 2:** Proteins unique to the HDL fraction.

Protein Accession	Protein Description	Plasma Avg. Scan Count ± SEM	Serum Avg. Scan Count ± SEM
P02763	Alpha-1-acid glycoprotein 1	7.3 ± 6.5	5.1 ± 4.7
P19652	Alpha-1-acid glycoprotein 2	2 ± 1.3	0.67 ± 0.41
P04217	Alpha- 1B-glycoprotein	5.1 ± 3.9	5.2 ± 4.3
P08697	Alpha-2-antiplasmin	2.6 ± 1.7	2.6 ± 1.9
P02750	Leucine-rich alpha-2-glycoprotein	0.89 ± 0.79	0.89 ± 1.2
P01011	Alpha-1-antichymotrypsin	6.9 ± 5.4	6.2 ± 6.7
P43652	Afamin	1.8 ± 1.3	1.7 ± 1.4
P35858	Insulin-like growth factor-binding protein	2.4 ±1	1.9 ± 1.2
P01019	Angiotensinogen	2.6 ± 1.9	1 ± 1.6
P01008	Antithrombin-III	5.2 ± 4.6	3.8 ± 4.2
P06727	Apolipoprotein A-IV	2.9 ± 2.8	3.4 ± 3.4
P02654	Apolipoprotein C-I	0.67 ± 0.41	1.4 ± 0.65
P02655	Apolipoprotein C-II	0.22 ± 0.25	0.44 ± 0.42
P05090	Apolipoprotein D	0.67 ± 0.5	0.44 ± 0.42
P02749	Apolipoprotein H	4.9 ±4	4.8 ± 3.8
O95445	Apolipoprotein M	0.89 ± 0.45	0.67 ± 0.5
O75882	Attractin	0.22 ± 0.25	0
P43251	Biotinidase	0.11 ± 0.19	0.22 ± 0.25
P08185	Corticosteroid-binding globulin	1 ± 0.9	0.78 ± 1
P00450	Ceruloplasmin	23.6 ± 7.6	23.1 ± 11.6
P00751	Complement factor B	4.4 ± 2.8	4.1 ± 3.3
P05156	Complement factor I	1 ± 0.9	1 ± 1.3
P06681	Complement C2	1.1 ± 0.67	0.67 ± 0.76
P01024	Complement C3	116 ± 43	139.6 ± 64 t
P01031	Complement C5	4.9 ± 2.7	4.1 ± 2.3
P13671	Complement component C6	3.3 ± 1.3	2.9 ± 1.6
P10643	Complement component C7	1.3 ± 0.87	1.6 ± 1.2
P07357	Complement component C8 alpha chain	0.22 ± 0.25	1 ± 0.87
P07358	Complement component C8 beta chain	1.2 ± 0.75	0.78 ± 0.81
P07360	Complement component C8 gamma chain	0.78 ± 0.48	0.78 ± 0.48
P02748	Complement component C9	0.78 ± 0.81	1.4 ± 1.5
P22792	Carboxypeptidase N subunit 2	0.56 ± 0.51	0.78 ± 0.56
Q16610	Extracellular matrix protein 1	0.44 ± 0.42	0.11 ± 0.19
P00748	Coagulation factor XII	1.2 ± 1.3	1.3 ± 1.1
P02765	Alpha-2-HS-glycoprotein	7.4 ± 6	6.1 ± 5.8
Q03591	Complement factor H-related protein 1	0.78 ± 0.63	0.56 ± 0.58
P06396	Gelsolin	3.6 ± 3.3	3.7 ± 4.6
P02790	Hemopexin	12.4 ± 9.9	12.9 ± 10.7
P05546	Heparin cofactor 2	1.2 ±1.3	1 ± 1
P04196	Histidine-rich glycoprotein	2.9 ±1.8	1.3 ± 1
P05155	Plasma protease C1 inhibitor	4.9 ± 2.5	4.6 ± 2.9
P19827	Inter-alpha-trypsin inhibitor heavy chain H1	7.4 ± 5.1	8.4 ± 5
Q06033	Inter-alpha-trypsin inhibitor heavy chain H3	1.1 ± 0.45	0.56 ± 0.42
Q14624	Inter-alpha-trypsin inhibitor heavy chain H4	16 ± 3.8	17.9 ± 9.8
P03952	Plasma kallikrein	1 ± 0.58	1.1 ± 0.67
P01042	Kininogen-1	5.3 ± 1.2	4.4 ± 2
P51884	Lumican	2 ± 0.65	2.4 ± 1.3
Q9NU22	Midasin	0.11 ± 0.19	0.11 ± 0.19
P36955	Pigment epithelium-derived factor	1 ±0.87	0.68 ± 1.2
Q96PD5	N-acetylmuramoyl-L-alanine amidase	1.4 ± 1.1	1.6 ± 1.5
P00747	Plasminogen	4.7 ± 5.1	6 ± 6.3
P02753	Retinol-binding protein 4	0.33 ± 0.29	0.44 ± 0.59
P35542	Serum amyloid A-4 protein	0.33 ± 0.5	0.22 ± 0.38
P02743	Serum amyloid P-component	2.4 ± 1.3	2.6 ± 1.4
P49908	Selenoprotein P	0.11 ± 0.19	0.22 ± 0.25
P04278	Sex hormone-binding globulin	0.22 ± 0.25	0.78 ± 0.9
Q8WYL5	Protein phosphatase Slingshot homolog 1	0.44 ± 0.42	0.11 ± 0.19

The average scan count ± SEM is provided for each protein identified in only the HDL fraction derived from both plasma and serum. (P < 0.001; * significantly greater in plasma; † significantly greater in serum)

**Table 3:** Proteins found in both the LDL fraction and the HDL fraction.

	LDL Fractions		HDL Fractions	
		
Protein Description	Plasma Avg. Scan Count ± SEM	Serum Avg. Scan Count ± SEM	Plasma Avg. Scan Count ± SEM	Serum Avg. Scan Count ± SEM
Alpha-1 -antitrypsin	2 ± 0.97	0.4 ± 0.52	39.8 ± 33.3	41 ± 43.8
Alpha-2-macroglobulin	475 ± 166	753 ± 171	11.1 ± 7.7	37.6 ± 38†
Serum Albumin	5.8 ± 5	7 ± 5.4	324.5 ± 243.4	306.1 ± 221.1
Alpha-1-microglobulin	0	0.4 ± 0.9	2.3 ± 1.1	2.3 ± 1.1
Apolipoprotein A-I	9 ± 2.2	11 ± 2.3	123.1 ± 30	107.2 ± 42
Apolipoprotein A-II	2.5 ± 1	3.2 ± 1	24.2 ± 6.5	26.7 ± 11.1
Apolipoprotein B-100	98 ± 19	167 ± 94†	0.44 ± 0.42	0.56 ± 0.30
Apolipoprotein C-III	5.7 ± 3.4	11.2 ± 8.8	1.9 ± 0.61	1.7 ± 0.58
Apolipoprotein E	4.8 ± 2.5	7.4 ± 2.7	1.8 ± 0.81	1.2 ± 1
Apolipoprotein-L1	2.3 ± 0.94	3 ± 1	0.78 ± 0.38	0.33 ± 0.29
Complement C1 s subcomponent	0.33 ± 0.3	0.8 ± 0.48	0.56 ± 0.3	0.78 ± 0.75
C4b-binding protein	17.5 ± 6.1	36 ± 28†	0	0.33 ± 0.41
Complement factor H	3. 5 ± 2.9	6.8 ± 6.1	5.7 ± 5.4	6.6 ± 4.7
Apolipoprotein J (Clusterin)	2.3 ± 0.79	2.8 ± 2.1	2.6 ± 0.65	2.9 ± 1.3
Complement C4-A	1.7 ± 1	0.2 ± 0.25	38.8 ± 14.7	34.2 ± 16
Coagulation factor XIII B chain	0.67 ± 0.47	0	0	0.44 ± 0.42
Fibrinogen alpha chain	133.7 ± 35.3 *	0	1.1 ± 1.1	0
Fibrinogen beta chain	149.2 ± 21 *	0	2 ± 1.7 *	0
Fibrinogen gamma chain	143 ± 28 *	0	0.78 ± 0.9	0
Fibronectin	57.2 ± 24	98.8 ± 38†	0.89 ± 0.79	0.44 ± 0.58
Haptoglobin	5.5 ± 4	19.2 ± 15.3†	11.3 ± 9.3	7 ± 4.1
Haptoglobin-related protein	19 ± 14	47.6 ± 33†	40.1 ± 28.8	29.4 ± 18
Inter-alpha-trypsin inhibitor heavy chain H2	0.5 ± 0.48	1.8 ± 1.25	7.6 ± 4.4	10.3 ± 6.8
Paraoxonase-1	0.5 ± 0.32	0.8 ± 1	2.7 ± 0.96	2.9 ± 1.1
Prothrombin	0	10.4 ± 2.1†	6.6 ± 4	4 ± 2.8
Vitronectin	0.5 ± 0.3	1.8 ± 1.1	4.1 ± 2.6	3.4 ± 1.7

The average scan count ± SEM is provided for each protein identified in both the LDL and HDL fractions derived from plasma and serum. (P < 0.001; * significantly greater in plasma; †significantly greater in serum)

### Analysis of LDL fraction

All of the proteins meeting the criteria for inclusion in the analysis of LDL fractions, along with the average scan counts for each protein, can be found in Additional File 2. Excluded from this list are two proteins which were identified as redundant proteins: pregnancy zone protein (PZP_HUMAN) and hemoglobin delta subunit (HBD_HUMAN). Among the proteins which were found to have significantly higher average scan counts in LDL particles derived from plasma are fibrinogens (alpha, beta and gamma chains), and hemoglobin subunits alpha and beta. Those proteins with significantly higher average scan counts found in the LDL fractions derived from serum include alpha-2-macroglobulin, apolipoprotein B-100, C4b-binding protein alpha chain, fibronectin, haptoglobin, haptoglobin-related protein and prothrombin. In total, 16 proteins were found in only the LDL fraction, and these proteins are listed in Table 1. Hemoglobin subunits alpha and beta are the proteins with significantly higher average scan counts in plasma, and none of the LDL-unique proteins exhibit significantly higher average scan counts in serum.

### Analysis of HDL fraction

All of the proteins meeting the criteria for inclusion in the analysis of the HDL fraction, along with the average scan counts for each protein, can be found in Additional File 3. Excluded from this list are two proteins which were identified as redundant proteins: pregnancy zone protein (PZP_HUMAN) and complement factor H related protein 1 (FHR1_HUMAN). Fibrinogen beta chain is the only protein that was found to have a significantly higher average scan count in plasma, while complement component 3 and alpha-2-macroblobulin are the proteins with significantly higher average scan counts in serum. Among the proteins unique to the HDL fraction, the only protein found with a significant difference between plasma and serum is complement component 3. A total of 65 proteins were identified to be unique to HDL, and these proteins are listed in Table 2.

## Discussion

The goal of the present study was to compare the utility of plasma and serum samples for shotgun lipoproteomic analyses. Over the past few years, a great deal of interest has been directed toward the elucidation of the proteins associated particularly with HDL particles as these particles have been found to contain a diverse protein constituent [3,5-7]. As alterations in particular proteins associated with HDLs have been identified in several disease states, there the potential for the discovery and detection of quantitative alterations in these proteins as indicators of early risk factors for cardiovascular disease. Advances in mass spectrometry have allowed for the global quantitative analysis of proteins in complex mixtures [16,17]. Since the quantitative aspect of such analyses provides great potential, it would be advantageous to select a sample which will yield optimal, unbiased results. To this end, as plasma and serum are the most commonly used samples for lipoprotein analyses, it was our objective to determine if the use of one sample type over the other would significantly affect the relative abundance of proteins. Furthermore, although proteomic analyses have been performed on LDL particles [4], we have not uncovered any shotgun proteomic studies on this lipoprotein class. To our knowledge, this is the first report of a shotgun proteomic analysis of the LDL fraction.

We identified approximately 65% of the proteins previously reported as LDL-associated proteins [4,18]. In our analysis, all of these confirmed LDL-associated proteins (alpha-1-antitrypsin, alpha-2-macroglobulin, serum albumin, apolipoproteins A-I, B-100, C-III, E, and J, fibrinogen, fibronectin and haptoglobin) were also identified in the HDL fraction (Table 2). In comparing our results to those of other mass-spectrometry-based lipoprotein analyses [3-7,18], this overlap between LDL- and HDL-associated proteins has been confirmed for all but one protein identified in our LDL fraction, fibronectin [which has previously been shown only to associate with LDL [4]].

With respect to the HDL fraction, we identified 65 total proteins unique to the HDL fraction. Of these 65 proteins, 15 (alpha-1-acid glycoprotein 1, alpha-1B-glycoprotein, alpha-2-antiplasmin, alpha-2-HS-glycoprotein, angiotensinogen, apolipoproteins C-I, D, and H, complement C3, complement C9, inter-alpha-trypsin inhibitor heavy chain H4, kininogen-1, retinol-binding protein 4, serotransferrin and transthyretin) were confirmed to be HDL-specific by other studies [3,5-7]. Four of the proteins listed in Table 2 (apolipoproteins A-IV, C-II and M, and serum amyloid A-4) were found by others to be associated with both HDL and LDL [3,5-7,18,19]. Taken together, these results indicate that there is still work to be done in order to conclusively state which proteins are associated uniquely with either LDL or HDL particles. If these lipoprotein particles do indeed share specific proteins, it will be of great interest to determine if these particles exchange certain proteins in the course of their normal biological function.

Regarding the differences between LDL particles and HDL particles derived from plasma and serum, our results indicate that the primary differences between the samples are found in the fibrinogen proteins which are contained within the fibrin clot and are removed from serum. Therefore, it is not surprising that the majority of the proteins exhibiting significantly higher scan counts in plasma are the fibrinogen proteins. With respect to LDL-associated proteins, it is of interest to note that apoB-100 was found at significantly higher levels in serum samples. This may suggest that if it is one's goal to analyze apoB-100, specifically, perhaps serum would be the preferred sample choice.

In addition to our findings related to the quantitative differences between LDL- and HDL-associated proteins derived from plasma and serum, the results from our shotgun proteomic analysis of LDL provide insights into the protein cargo associated with these particles. All of the proteins listed in Table 1, which we have determined to be unique to the LDL fraction derived from our FPLC isolation, have never been reported as LDL-associated proteins. These findings suggest that we have identified several potentially novel, LDL-associated proteins. However, given the fact that the majority of these proteins are found at relatively low levels, future studies are warranted in order to confirm their association. Nonetheless, proteins such as galectin-3-binding protein and several complement subcomponents may provide insight into the biological function of LDL. It has been reported that HDL particles are enriched in several complement proteins, suggesting that HDLs play a role in complement pathway activation [7]. As we were able to identify several different complement subcomponents in the LDL fraction, this may suggest that LDL particles also play a role in complement pathway activation. Of other potential interest is galectin-3-binding protein, which has been shown to promote integrin-associated cell adhesion [20,21]. Given the critical role of cell adhesion contributing to plaque formation in atherosclerosis and the central role that LDL particles play in the progression of this disease, a protein such as galectin-3-binding protein may serve as the basis for future studies regarding its possible etiological implication.

A concern with studies such as this is with the purity of the lipoprotein isolations and whether or not certain identified proteins are truly lipoprotein-associated, or are contaminants from either plasma or serum which happen to co-elute with either LDL or HDL particles in the FPLC-SEC fractionation. Our study may suggest that an identification of a particular protein in either LDL or HDL fractions derived from both serum and plasma samples bolsters the argument that it is indeed associated with a lipoprotein particle and not merely a contaminant from either plasma or serum. For example, if certain proteins, such as the fibrinogen proteins, were found at significantly higher levels in just plasma- or serum-derived lipoprotein fractions, it would suggest that those proteins are likely contaminants and may not in fact be associated with a lipoprotein particle. Overall, our data suggests that a successful quantitative analysis of either the LDL or HDL proteomes can be accomplished with either plasma or serum samples.

## Methods

### Plasma and Serum Samples

The protocol for the acquisition of blood samples from volunteers was approved by an Institutional Review Board at the Medical College of Wisconsin. Written consents were obtained from each volunteer prior to inclusion in this study. Plasma and serum samples were obtained from three random, healthy donors. No identifying health information was collected. For the methods described below, for both the plasma and serum samples, a total of three biological replicates were analyzed.

### Materials

All chemicals were purchased from Sigma-Aldrich (St. Louis, MO) unless otherwise noted.

### Isolation of plasma lipoproteins by FPLC-SEC

FPLC-size exclusion chromatography (FPLC-SEC) analysis was carried out on a BioLogic DuoFow QuadTec 10 System equipped with a BioFrac fraction collector (Bio-Rad Laboratories, Inc., Hercules, CA) as described by [14]. Briefly, a single Superdex 200 10/300 GL column (GE Healthcare, Uppsala, Sweden) was used for the size-exclusion chromatography. Elutions were performed in a 1 mM EDTA, 150 mM NaCl, 0.02% NaN_3 _phosphate buffered saline solution, pH 7.4. Prior to each sample injection into the FPLC-SEC system, 12 mL of eluent buffer, run at 0.3 mL/min, was used to ensure equilibration of the column. Plasma and serum sample aliquots of 200 μL were used for each injection. The elutions were carried out at a flow rate of 0.30 mL/min with a maximum pressure of 218 psi. Fractions of 0.5 mL were collected in pre-treated polypropylene tubes throughout the analysis. Fractions corresponding to discrete elution peaks and/or troughs were pooled as previously described [14] and then concentrated using pre-blocked Vivaspin 500 spin columns (Vivascience, Hannover, Germany). After pooling the 0.5 mL fractions together based on their correspondence with either a discrete elution peak or trough, a total of eight different fraction sets is formed. Fraction sets 2-4, which elute between approximately 20 and 23.5 mL, corresponds to the LDL fraction. These volumes take into account the 12 mL used for column equilibration. Fraction sets 4-7, which elute between approximately 23.5 and 28 mL, contain the HDL fraction. Extensive studies were previously performed by our group in order to validate the location of both LDL and HDL within these elution volumes [14]. As indicated, there is a potential overlap between the LDL and HDL fraction sets in fraction set four. In order to avoid potential contamination and crossover between the LDL and HDL proteomic analysis, fraction set four, which elutes between 23.5 and 24.5 mL, was not included.

### Chloroform extractions and sample preparation for mass spectral analysis

The concentrated FPLC-SEC plasma and serum fraction sets were delipidated via a series of chloroform extractions [protocol adapted from Mirza *et al*. [22] and previously described by Collins *et al*. [14]]. Following the delipidation procedure, the proteins were reduced with 10 mM dithiothreitol (DTT) at 37°C for 30 min and subsequently alkylated with 55 mM iodoacetamide [23] at 37°C for 45 min in dark. The proteins were then digested with trypsin (Promega, Madison, WI) at a ratio of 1:50 trypsin to protein for 12 h at 37°C. Digestions were quenched with the addition of 1 μL of 10% formic acid. The samples were then cleaned with C_18 _Zip-Tips (Millipore, Billerica, MA) prior to mass spectral analysis.

### Nano-HPLC-electrospray ionization tandem mass spectrometry

The protein digests from both plasma and serum lipoprotein fractions were analyzed on a ThermoFinnigan LTQ ion trap mass spectrometer interfaced with a nano-LC system (Thermo Electron) equipped with an autosampler through which samples were loaded onto a C_18 _capillary column (100 × 0.1 mm). The capillary column was packed in-house with 5 μm C_18 _RP particles (Phenomenex, Cheshire, UK). The solvents A and B, used for the chromatographic separation of peptides, were 5% acetonitrile in 0.1% formic acid and 95% acetonitrile in 0.1% formic acid, respectively. The protein digest injected onto the microcapillary column was resolved at a rate of 150 μL/min, by the following gradient conditions: 0-120 min 0-25% B, 120-180 min 25-75% B, 180-190 min 75-100% B, 190-200 min 100% B, 215-300 min 100% A.

### Protein identification and data analysis

Peak lists were generated from raw LC-MS/MS spectra using ExtractMS v. 3. The raw data acquired by the mass spectrometry experiment were searched against the human UniProt database (Uniprot v. 49.1, which contains 13,488 proteins and 7,360,189 amino acids) using the SEQUEST [24] (TurboSEQUEST v. 27 rev. 12) search engine. Additional search parameters included a precursor-ion mass tolerance of ±2.5, and a fragment-ion mass tolerance of 0. The output files from the database search were filtered and summarized using the program Epitomize [25]. The filter allows for a positive peptide, and hence protein, identification based on Protein Probability scores. The algorithm which generates the Protein Probability scores utilizes decoy database searching and is a modified version of the Peptide Prophet [19] algorithm. The Protein Probability scores are calculated with a modified version of the algorithm, Peptide Prophet.

Visualize http://proteomics.mcw.edu/visualize, a bioinformatics tool which allows for visualization and analysis of data produced by Epitomize, allows for the combination and comparison of the data obtained from each of the plasma and serum sample runs for each fraction set. Visualize allows the user to manually set the criteria for filtering data. The criteria for the positive identification of a protein, in this case, were set at a protein probability of 0.85 and a requirement for the identification of at least two unique peptides. These criteria are equivalent to a global false discovery rate of five percent. Due to their ubiquitous presence in both plasma and serum, and lack of evidence that they contribute meaningfully to lipoprotein function, immunoglobulins were not analyzed. Keratin proteins were also removed from the data set as they are common contaminants of such experiments. In order to address the issue of protein name redundancy, we not only searched our mass spectrometry data against a non-redundant protein database, UniProt, but we also utilized a function in Visualize which allows for the assessment of protein redundancy. Using this feature of Visualize, we performed a removal of any redundant proteins at the peptide level. An average scan count and standard error of the mean was calculated for each protein derived both from plasma and from serum for both the LDL and HDL fractions. P values were calculated for each protein and then corrected by a *Bonferroni *adjustment procedure. These calculations allow us to compare and contrast the protein population associated with LDLs and HDLs derived from plasma and serum.

## List of Abbreviations

LDL: low-density lipoprotein; HDL: high-density lipoprotein; FPLC-SEC: fast protein liquid chromatography-size exclusion chromatography; PON-1: paraoxonase-1; C3: complement component 3; SAA: serum amyloid A; HUPO: human proteome organization; PPP: Plasma Proteome Project; APOB-100: apolipoprotein B-100; apoA-I: apolipoprotein A-I;

## Competing interests

The authors declare that they have no competing interests.

## Authors' contributions

LAC participated in the design of the study, acquisition of samples, carried out the FPLC-SEC, mass spectral and data analyses, and drafted the manuscript. MO conceived of the study, and participated in the interpretation of the results, its design and coordination, and in drafting the manuscript. All authors read and approved the final manuscript.

## Supplementary Material

Additional file 1 **Supplemental Figure 1**. A total ion chromatogram from one FPLC-derived plasma HDL fraction and the mass spectrum for a selected peptide of the apoliproptein A-I protein. Click here for file

Additional file 2 **Supplemental Data Table 1**. Average scan counts for each protein identified in either plasma or serum for LDL fraction sets in 3 controls. Click here for file

Additional file 3 **Supplemental Data Table 2**. Average scan counts for each protein identified in either plasma or serum for HDL fraction sets in 3 controls. Click here for file
