# Supplementary material for: Quantitative comparison of lipoprotein fractions derived from human plasma and serum by liquid chromatography-tandem mass spectrometry
Source: Proteome Sci. 2010 Jul 29;8:42. doi: 10.1186/1477-5956-8-42 (PMC2918550; doi:10.1186/1477-5956-8-42)
Supplement: Additional file 1 — Supplemental Figure 1. A total ion chromatogram from one FPLC-derived plasma HDL fraction and the mass spectrum for a selected peptide of the apoliproptein A-I protein. [file 1477-5956-8-42-S1.DOC]

**Supplemental Data Figure:** **A total ion chromatogram from one FPLC-derived plasma HDL fraction and the mass spectrum for a selected peptide of the apoliproptein A-I protein.** **(a)** Total ion chromatogram from one of the FPLC-derived plasma HDL fractions. **(b)** Mass spectrum assigning b- and y-ions for a peptide of the given sequence from apolipoprotein A-I.
