# Supplementary material for: Quantitative comparison of lipoprotein fractions derived from human plasma and serum by liquid chromatography-tandem mass spectrometry
Source: Proteome Sci. 2010 Jul 29;8:42. doi: 10.1186/1477-5956-8-42 (PMC2918550; doi:10.1186/1477-5956-8-42)
Supplement: Additional file 2 — Supplemental Data Table 1. Average scan counts for each protein identified in either plasma or serum for LDL fraction sets in 3 controls. [file 1477-5956-8-42-S2.DOC]

**Supplemental Data Table 1: Average scan counts from FPLC-derived plasma and serum LDL fraction sets in three different individuals.** The average scan count ± standard error of the mean (SEM) is provided for each protein identified in the LDL fraction derived from both plasma and serum. (P < 0.001; * significantly higher in plasma; † significantly higher in serum).
